# Magnetic and Electronic Properties of Sr Doped Infinite-Layer NdNiO_2_ Supercell: A Screened Hybrid Density Functional Study

**DOI:** 10.3390/molecules28103999

**Published:** 2023-05-10

**Authors:** Yawen Hua, Meidie Wu, Qin Qin, Siqi Jiang, Linlin Chen, Yiliang Liu

**Affiliations:** College of Electronics and Information & Key Lab of Information Materials of Sichuan Province & Key Laboratory of Electronic and Information Engineering, State Ethnic Affairs Commission, Southwest Minzu University, Chengdu 610041, China

**Keywords:** Nd_9*-n*_Sr*_n_*Ni_9_O_18_ (*n* = 0–2), magnetism, electronic structure, HSE06

## Abstract

To understand the influence of doping Sr atoms on the structural, magnetic, and electronic properties of the infinite-layer NdSrNiO_2_, we carried out the screened hybrid density functional study on the Nd_9-*n*_Sr*_n_*Ni_9_O_18_ (*n* = 0–2) unit cells. Geometries, substitution energies, magnetic moments, spin densities, atom- and lm-projected partial density of states (PDOS), spin-polarized band structures, and the average Bader charges were studied. It showed that the total magnetic moments of the Nd_9_Ni_9_O_18_ and Nd_8_SrNi_9_O_18_ unit cells are 37.4 and 24.9 emu g^−1^, respectively. They are decreased to 12.6 and 4.2 emu g^−1^ for the Nd_7_Sr_2_Ni_9_O_18_-Dia and Nd_7_Sr_2_Ni_9_O_18_-Par unit cells. The spin density distributions demonstrated that magnetic disordering of the Ni atoms results in the magnetism decrease. The spin-polarized band structures indicated that the symmetry of the spin-up and spin-down energy bands around the Fermi levels also influence the total magnetic moments. Atom- and *lm*-projected PDOS as well as the band structures revealed that $\mathrm{Ni}(d_{x^{2}-y^{2}})$ is the main orbital intersecting the Fermi level. As a whole, electrons of Sr atoms tend to locate locally and hybridize weakly with the O atoms. They primarily help to build the infinite-layer structures, and influence the electronic structure near the Fermi level indirectly.

## 1. Introduction

Superconductivity has attracted significant interest both for exploring fundamental science and for a wide range of technological applications, such as energy storage, transportation, medical imaging, and power-generation systems [1]. Since the discovery of high critical temperature superconductivity in La_1.85_Ba_0.15_CuO_4_ by Bednorz and Müller in 1986 [2], the superconducting mechanism has not achieved consensus in spite of considerable quantities of experimental and theoretical studies [3]. In recent years, the discovery of a new class of superconductivity in infinite-layer nickelate has attracted tremendous attention in the superconductivity area [4,5,6,7,8,9,10,11], which has sparked researchers’ expectations of addressing this issue. That is because the novel Sr-doped NdNiO_2_ superconductors were assumed as a structural and electronic analog of the well-known cuprate superconductor [12,13,14]. Specifically, despite certain differences [15,16], both Ni^+^ and Cu^2+^ ions are formally *d*^9^ in the respective parent compounds. Near the Fermi level, Ni and Cu also have similar electronic structures. Thereby, a study on the nickelate superconductors would have a great impact on not only the nickelates but also the other layered transition-metal oxides [17,18,19]. It may provide a new opportunity for further understanding the unconventional superconductivity.

Superconductivity in nickelates also poses a challenge to the proposed explanations. Unlike conventional superconductivity, electron-phonon coupling had been ruled out from the superconducting mechanism of nickelate [15,20,21,22]. Instead, a large number of standpoints from different perspectives had been proposed. Li et al. argued that the superconductivity may not be originated from the expected Nd_0.8_Sr_0.2_NiO_2_, but arise from the interface or the stress effect [23]. However, based on the comparison between NdNiO_2_ and Nd_0.8_Sr_0.2_NiO_2_, D. Li’s opinion was that the interface effect alone does not lead to superconductivity [4]. Theoretical studies by Si et al. indicated that the unexpected topotactic hydrogen intercalation has dramatic consequences for the superconductivity of the infinite-layer Sr-doped NdNiO_2_ [12]. Malyi et al. even emphasized that stoichiometric NdNiO_2_ is significantly unstable, and the incorporation of hydrogen can reduce its instability [24].

In spite of some conflicting viewpoints [16,25,26,27], magnetic and electronic properties are widely believed to be the key factors to comprehend superconductivity [6,28,29,30,31]. From these perspectives, considerable quantities of viewpoints on Nd_0.8_Sr_0.2_NiO_2_ has also been set in [21,23,32,33,34]. Regarding the magnetism being different from the NdNiO_3_ and cuprate, antiferromagnetic coupling in nickelates is substantially weaker [4,5,15,16,32]. That was attributed to the competition between ferromagnetic (FM) and antiferromagnetic (AFM) exchanges, which significantly suppressed the magnetic tendency [3]. LDA + DMFT studies by Ryee et al. indicated that hole-doping induced magnetic two-dimensionality is the key to superconducting Nd_1−*x*_Sr*_x_*NiO_2_ [35]. Zhang et al. supported that the self-doping effect, namely strong Kondo coupling-induced holon-doublon excitations, suppresses the antiferromagnetic (AF) long-range order and produces the paramagnetic metallic ground state even in the parent compound NdNiO_2_. Sr doping introduces extra holes on the Ni sites, which further drives the system away from the AF Mott insulating phase [32]. A multiorbital description within the spin-freezing theory was employed by Werner et al. They concluded that local moment fluctuations rather than antiferromagnetic fluctuations induce the pairing mechanism of nickelate superconductors [15,36].

With respect to the electronic structure, nickelates were suggested as unconventional superconductivity with most likely a *d*-wave order parameter [37,38]. Near the Fermi level, the electronic structure is dominated by $\mathrm{Ni}(d_{x^{2}-y^{2}})$ orbitals [5,14,20,37,38,39,40], and the localized $\mathrm{Ni}(d_{x^{2}-y^{2}})$ band is self-doped with holes by the low density Nd(5*d*) conduction electrons [5,16,19,30,33,35,36,37,38,39]. The presence of the 5*d* states at the Fermi surface leads to speculation about an important role of the hybridization between the strongly correlated Ni(3*d*) and more extended Nd(5*d*) states. However, different viewpoints were also proposed. With a three-orbital model, Nomura et al. indicated that NdNiO_2_ hosts an almost isolated $\mathrm{Ni}(d_{x^{2}-y^{2}})$ orbital system, namely its hybridization with the states in the Nd layer is tiny [20].

In this paper, four models with increasing Sr-doping proportion were built to study the effect of the doping Sr atoms on the magnetic and electronic properties of the infinite-layer NdNiO_2_. Compared with the molecular dynamics simulations [41,42], the static hybrid density functional method was employed to calculate the geometrical structures, stabilities, magnetism, electronic structures, and charges of the Nd_9−*n*_Sr*_n_*Ni_9_O_18_ (*n* = 0–2) unit cells. The results were discussed and analyzed in Section 2. Brief conclusions were given in the last Section.

## 2. Results and Discussions

### 2.1. Structures and Stabilities

**Figure 1:**
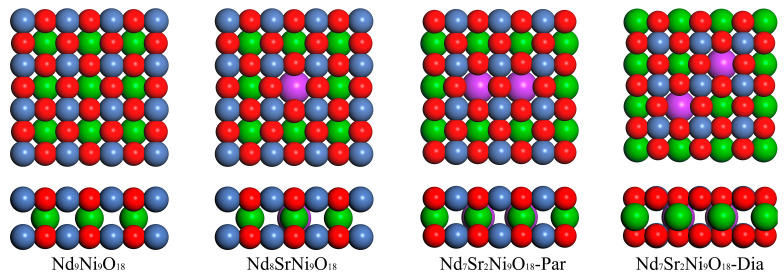
Unit cells of the Nd_9-*n*_Sr*_n_*Ni_9_O_18_ (*n* = 0–2). The red, green, blue, and purple balls denote O, Nd, Ni, and Sr atoms, respectively.

The optimized models of the Nd_9-*n*_Sr*_n_*Ni_9_O_18_ (*n* = 0–2) are shown in Figure 1. Figure 1 displays the top views (upper diagram) and side views (lower diagram) of Nd_9_Ni_9_O_18_, Nd_8_SrNi_9_O_18_, Nd_7_Sr_2_Ni_9_O_18_-Par, and Nd_7_Sr_2_Ni_9_O_18_-Dia unit cells, respectively. Among them, Nd_9_Ni_9_O_18_ is the pure nickelate with no doping Sr ions. Nd_8_SrNi_9_O_18_ is endowed with one Sr ion. Nd_7_Sr_2_Ni_9_O_18_-Par and Nd_7_Sr_2_Ni_9_O_18_-Dia denote two inequivalent Nd_7_Sr_2_Ni_9_O_18_ models. One possesses parallel aligned Sr ions, the other has diagonally aligned Sr ions with the lattice vector. Calculated lattice constants of the Nd_9_Ni_9_O_18_ supercell are *a* = *b* = 11.73 Å (3.91 Å for single cell) and *c* = 3.31 Å, which consist well with the experimental results of *a* = *b* = 3.92 Å and *c* = 3.31 Å [34]. With respect to the Nd_8_SrNi_9_O_18_ and Nd_7_Sr_2_Ni_9_O_18,_ the *a* and *b* stay almost invariable, whereas the *c* axes rise to 3.32 and 3.34 Å consecutively. The value 3.34 Å also matches well with the experimental value 3.37 Å (3.34~3.38 Å) of Nd_0.8_Sr_0.2_NiO_2_ film [4]. That is to say, the more Nd atoms were replaced, the longer the *c* axis is. 

The lattice energies of these models also increase gradually with the Sr doping. The substitution energy of a single Sr atom was calculated with the following formula [43,44].

(1) $$E_{S1}=E_{T}[{\mathrm{Nd}}_{8}{\mathrm{SrNi}}_{9}O_{18}]-E_{T}[{\mathrm{Nd}}_{9}{\mathrm{Ni}}_{9}O_{18}]-(\mu_{\mathrm{Sr}}-\mu_{\mathrm{Nd}})$$

The substitution energy of the second Sr atom is,

(2) $$E_{S2}=E_{T}[{\mathrm{Nd}}_{7}{\mathrm{Sr}}_{2}{\mathrm{Ni}}_{9}O_{18}]-E_{T}[{\mathrm{Nd}}_{8}{\mathrm{SrNi}}_{9}O_{18}]-(\mu_{\mathrm{Sr}}-\mu_{\mathrm{Nd}})$$

where $E_{T}$ denotes the total energy of the corresponding system. The $\mu_{\mathrm{Sr}}$ and $\mu_{\mathrm{Nd}}$ are chemical potentials for Sr and Nd, respectively. The $\mu_{\mathrm{Sr}}$ was calculated as the total energy per atom in the face-centered cubic Sr, and the $\mu_{\mathrm{Nd}}$ was calculated as the total energy per atom in the hexagonal close-packed Nd. As a result, the calculated substitution energy of the first Sr atom is 1.04 eV. Thereby, the substitution of a Sr atom requires extra energies, in other words, the doped architectures are less energetically stable than the pure NdNiO_2_. The substitution energy of the second Sr atom is 1.03 eV and 0.69 eV for Nd_7_Sr_2_Ni_9_O_18_-Par and Nd_7_Sr_2_Ni_9_O_18_-Dia, respectively. In comparison with the first Sr atom, the value of the second Sr atom for the Nd_7_Sr_2_Ni_9_O_18_-Par is almost invariable, while for the Nd_7_Sr_2_Ni_9_O_18_-Dia structure, the substitution energy decreases apparently. That is, the alignment of the Sr atoms influenced the stabilities. The two Sr atoms are more prone to aligning along the diagonal line instead of the parallel line of the lattice. 

### 2.2. Magnetism

The NdNiO_2_ supercell was delegated to test the spin-orbital coupling effect first. The <0 0 1> and <0 1 0> spin quantization axes were taken into account. Only small energy differences of 0.01 eV were found for these two directions. Except for that, the obtained magnetic moments for both cases were (0, 0, 9) $\mu_{b}$, which resembled the collinear calculation. Therefore, the spin-orbital coupling effect was omitted, and collinear magnetic structures were calculated in this study. 

**Figure 2:**
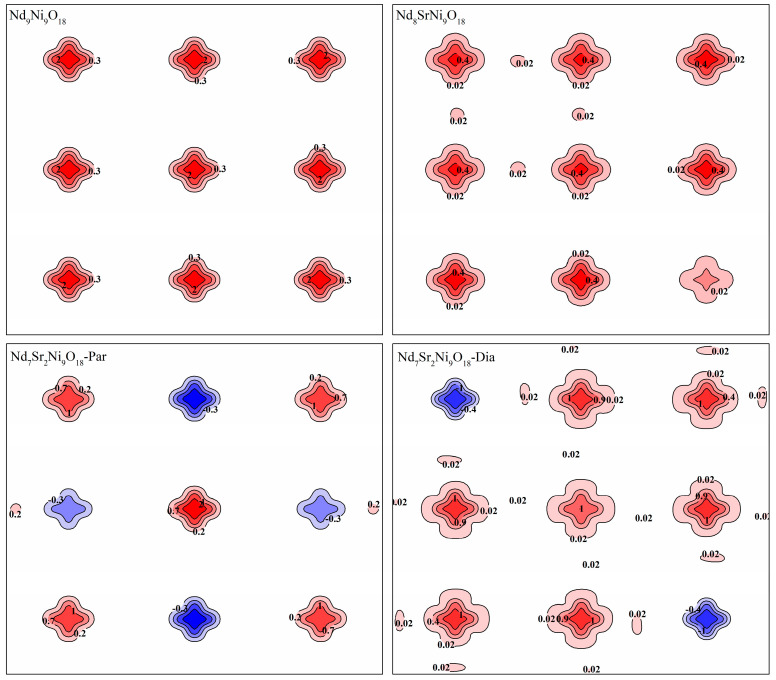
Spin densities contour for the Nd_9-*n*_Sr*_n_*Ni_9_O_18_ (*n* = 0–2) (001) surface. The blue and red fillings represent negative and positive electron spin densities. The unit of the contour is e.

Spin density maps in Figure 2 show a rich visualization of the spin density distributions at (001) surface defined by the [100] and the [001] direction. Magnetic moments of all the models arranged themselves in the two-dimensional NiO_2_ planes and mainly localized on the Ni atoms. 

In accordance with the experimental observations [4,16,34,45,46], no sign of apparent magnetic ordering was seen in the considered models. Especially, unlike the superconducting cuprate, there is no evidence of long-range antiferromagnetic order exhibited, which was also not observed by Fowlie et al. with muon spin rotation/relaxation experiment and Chen et al. with the quantum Monte Carlo method [47,48]. The Nd_9_Ni_9_O_18_ displays mostly a paramagnetic ground state. Obtained total magnetic moment of the Nd_9_Ni_9_O_18_ unit cell is 37.4 emu g^−1^ (~1 $\mu_{b}$ per Ni ion). It is also comparable with the experimental data of 26.7 emu g^−1^ at ~2 K and 30 kOe [23]. With the Sr substitution for Nd atoms, magnetic disordering namely, a slow fluctuation of the magnetic moment appears [15]. Via a two-orbital model, Hu et al. interpreted this as the competition between ferromagnetism and anti-ferromagnetism exchanges, thus the magnetic tendency is significantly suppressed [3]. The magnetic moment of the Nd_8_SrNi_9_O_18_ shrinks to 24.9 emu g^−1^. For the Nd_7_Sr_2_Ni_9_O_18_, spins of certain Ni atoms turn negative. Total magnetic moments of the Nd_7_Sr_2_Ni_9_O_18_-Dia and Nd_7_Sr_2_Ni_9_O_18_-Par models decrease furtherly to 12.6 and 4.2 emu g^−1^, respectively. The calculated value of 12.6 emu g^−1^ for Nd_7_Sr_2_Ni_9_O_18_-Dia is in concordance with the experimental value of 19.1 emu g^−1^ for Nd_0.8_Sr_0.2_NiO_2_ [23]. The unfounded value of 4.2 emu g^−1^ may be due to the higher energy of Nd_7_Sr_2_Ni_9_O_18_-Par than the Nd_7_Sr_2_Ni_9_O_18_-dia. In other words, the fabrication of Nd_7_Sr_2_Ni_9_O_18_-Dia is easier than the Nd_7_Sr_2_Ni_9_O_18_-Par during the experimental assembly. The decreasing tendency of magnetic moments demonstrated that doping of Sr elements obviously suppress the magnetic moment of NdNiO_2_ [23]. 

### 2.3. Electronic Structure

**Figure 3:**
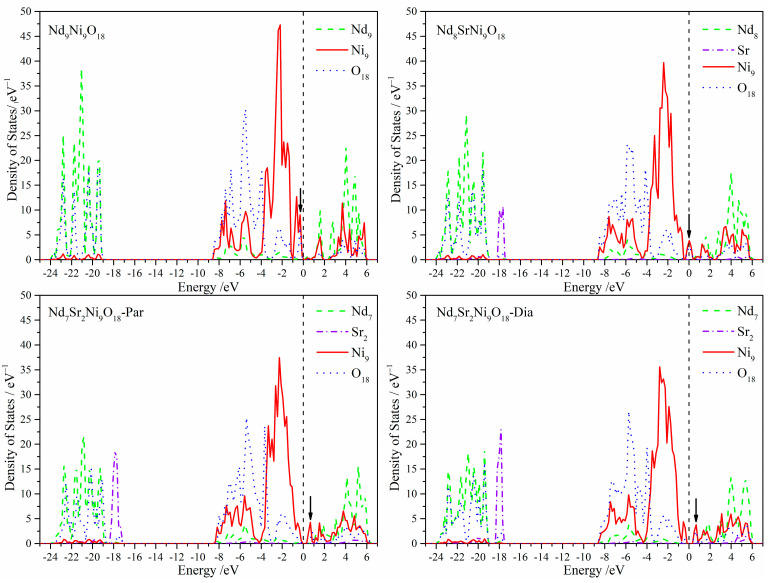
Site-projected partial density of states for the Nd_9-*n*_Sr*_n_*Ni_9_O_18_ (*n* = 0–2) models. The vertical dashed line denotes the Fermi level. The arrows underline the apparent variation near the Fermi level.

Atom-projected partial density of states (DOS) for the Nd_9−*n*_Sr*_n_*Ni_9_O_18_ (*n* = 0–2) models are listed in Figure 3. The Fermi levels read as 0 eV. On one hand, for the Sr substituted models, electrons of Sr atoms tend to locate locally at about −18 eV and hybridize weakly with the O atoms. Except for that, no conspicuous hybridization was found among Sr atoms and the other atoms. Apart from the O–Sr hybridization, the primary hybridization can be classified approximately into two energy ranges, namely, −24~−19 eV for O–Nd atoms and −9~0 eV for the O–Ni atoms. Further analysis based on our *lm-*projected DOS illuminated them as the $O\left(s\right)\text{-}\mathrm{Sr}\left(p_{x},p_{y},p_{z}\right)$,$O\left(s\right)\text{-}\mathrm{Nd}\;\left(p_{x},p_{y},p_{z}\right)$, and the $O\left(p_{x},p_{y},p_{z}\right)\text{-}\mathrm{Ni}\left(d_{xy},d_{yz},d_{xz},d_{z^{2}},d_{x^{2}-y^{2}}\right)$ orbital overlaps. Hybridization between the Ni electronic states and the states of the Nd layer is tiny [20].

**Figure 4:**
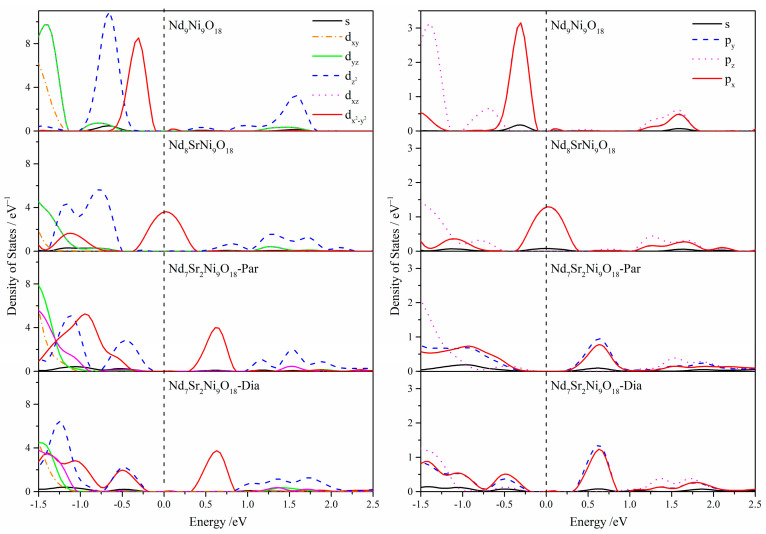
The *lm*-projected partial density of states for the Ni (**left**) and O (**right**) atoms of the Nd_9-*n*_Sr*_n_*Ni_9_O_18_ (*n* = 0–2) models.

On the other hand, in addition to a slight number of Nd orbitals, electrons near the Fermi levels primarily belong to the Ni and O atoms. It should be noticed that Sr atoms influence mainly the electronic structures near the Fermi levels. The most apparent variation is the peaks marked by arrows, which shift right with the increasing Sr atoms. To clarify further which orbitals contribute to the peaks, the *lm*-projected DOS for Ni and O atoms are shown in Figure 4. It is quite clear that the peaks originate from the $\mathrm{Ni}\;\left(d_{x^{2}{-y}^{2}}\right)\text{-}O\;\left(p_{x},\;p_{y}\right)$ orbital hybridization which is taken as the base of superconductivity. That is, doping of Sr atoms results in the uplift of the $\mathrm{Ni}\;\left(d_{x^{2}{-y}^{2}}\right)$ and $O\;\left(p_{x},\;p_{y}\right)$ orbitals around the Fermi levels. They also show that the $\mathrm{Ni}\;\left(d_{z^{2}}\right)$ tend to broaden and move toward the Fermi level.

**Figure 5:**
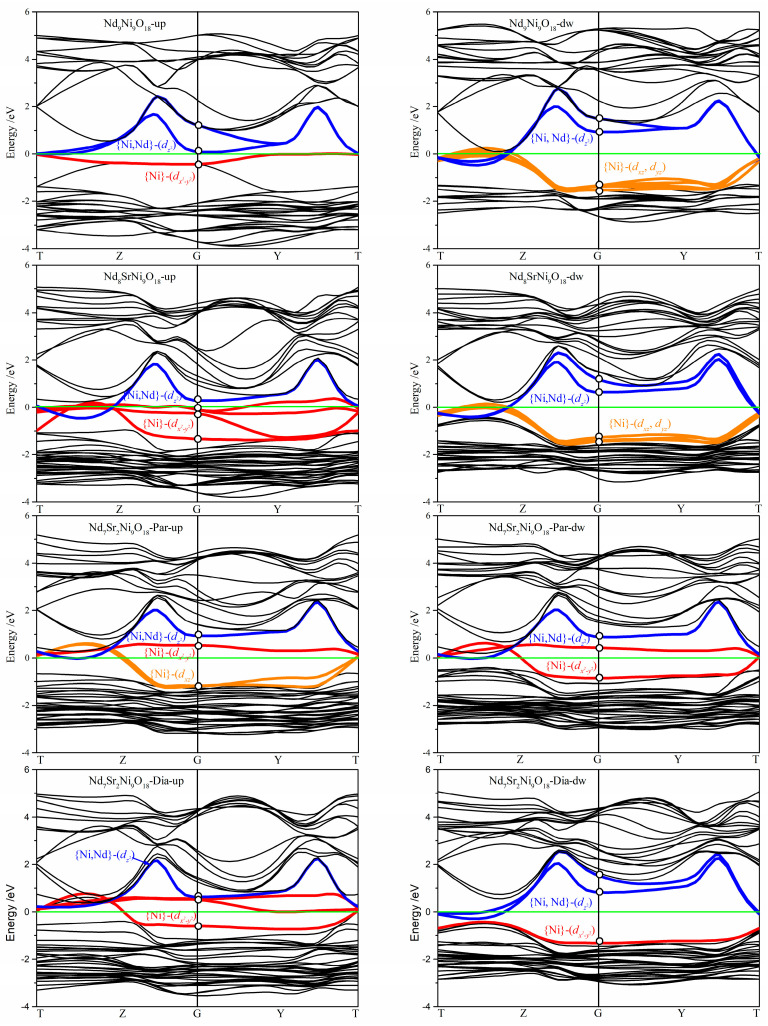
Spin-up (**left**) and spin-down (**right**) band structures for the Nd_9-*n*_Sr*_n_*Ni_9_O_18_ (*n* = 0–2) models. The green horizontal solid line denotes the Fermi level. The black lines represent the bands being away from the Fermi levels. The orange, red, and blue lines correspond to the $\mathrm{Ni}(d_{xz},d_{yz})$, $\mathrm{Ni}(d_{x^{2}-y^{2}})$ and $\{\mathrm{Ni},\mathrm{Nd}\}\text{-}(d_{z^{2}})$ orbitals intersecting the Fermi levels, respectively.

Spin-polarized band structures of the Nd_9-*n*_Sr*_n_*Ni_9_O_18_ (*n* = 0–2) models are listed in Figure 5. The bands intersecting the Fermi levels were marked with orange, red, and blue. Combing with the *lm*-decomposed calculations for each ion at each K-points and based on the analysis at G (0, 0, 0) point, it found that these three colored bands corresponded to the $\mathrm{Ni}(d_{xz},d_{yz})$, $\mathrm{Ni}(d_{x^{2}-y^{2}})$ and $\{\mathrm{Ni},\mathrm{Nd}\}\text{-}(d_{z^{2}})$ orbitals, respectively. That is, the band couple primarily acts as the hybridization among these three kinds of orbitals near the Fermi level. The $\{\mathrm{Ni},\mathrm{Nd}\}\text{-}(d_{z^{2}})$ orbitals close to the Fermi level were also discovered by Tam et al. using NiL_3_ resonant X-ray scattering [49]. Moreover, $\mathrm{Ni}(d_{x^{2}-y^{2}})$ is the main orbital intersecting the Fermi level. 

With the substitution of Sr atoms, the $\mathrm{Ni}(d_{x^{2}-y^{2}})$ bands tend to widen. Being in accordance with the DOS, they rise gradually and pass the Fermi level. Apart from the $\mathrm{Ni}(d_{x^{2}-y^{2}})$ bands, some $\{\mathrm{Ni},\mathrm{Nd}\}\text{-}(d_{z^{2}})$ bands also intersect the Fermi levels. However, these bands favor being above the Fermi level and slightly higher than the $\mathrm{Ni}(d_{x^{2}-y^{2}})$ bands. In one word, energy order is generally arranged as follows: $\{\mathrm{Ni},\mathrm{Nd}\}\text{-}(d_{z^{2}})>\mathrm{Ni}(d_{x^{2}-y^{2}})>\mathrm{Ni}(d_{xz},d_{yz})$. Overlap of the $\mathrm{Ni}(d_{z^{2}})$ and $\mathrm{Nd}(d_{z^{2}})$ bands as well as their intersection with the bands was interpreted as the Kondo coupling of Ni(3*d*) and Nd(5*d*) electrons by Zhang et al. [32]. Comparing the spin-up and –down bands around the Fermi level for the four Nd_9-*n*_Sr*_n_*Ni_9_O_18_ (*n* = 0–2) models, it was also found that the symmetry of the bands has an impact on the magnetic moments. The highest symmetry of the spin-up and spin-down energy bands for the Nd_7_Sr_2_Ni_9_O_18_-Par lead to the smallest magnetic moment. While the lowest symmetry of the spin-up and spin-down energy bands for the Nd_9_Ni_9_O_18_ induces the biggest magnetic moment. Considering the DOS and Energy bands comprehensively, Sr atoms influence the electronic structure indirectly. 

### 2.4. Charge

According to the Hall coefficient measure, Nd_0.8_Sr_0.2_NiO_2_ was reported as the hole-doped superconductor [4]. But what doped holes are introduced upon the chemical substitution of the Sr atom, the Ni or O sites? Hirsch et al. assumed that, like the cuprates, the added holes go into the oxygen pπ orbitals [50]. Whereas, Zhang and Chang et al. suggested that the doped holes enter the Ni orbitals and form a conducting band [15,32,51].

In this work, Bader charge analysis was imposed on the Nd_9-*n*_Sr*_n_*Ni_9_O_18_ (*n* = 0–2) models. Average Bader charges for each kind of atom were listed in Table 1. The involved numbers of valence electrons for the isolated Nd, Sr, Ni, and O atoms are 11, 10, 10, and 6, respectively. As can be seen, the average charge of the Ni atom is approximately +1, which agrees with the widely accepted opinion [3,52]. Both the Ni and O atoms lose electrons with the increasing substitution of Sr atoms. Specifically, the average numbers of electrons decrease from 7.39 to 7.33 and 7.36 for the O atoms. The average numbers of electrons decrease from 9.29 to 9.16 and 9.18 for Ni atoms. However, for the two Nd_7_Sr_2_Ni_9_O_18_ models, the Ni atoms nearest both the two Sr atom favor getting the minimums of 9.08 (Dia) and 9.13 (Par).

## 3. Models and Computational Details

The geometries of the infinite-layer nickelates resemble those of the cuprates. Concretely, the parent compound NdNiO_2_ possesses a space group crystalline symmetry of P4/mmm. The Ni-site surrounded by four O anions establishes a planar square NiO_2_ structure [3,53]. Each Ni ion is surrounded by four O ions in the basal plane and is absent of apical oxygens above and below it. These NiO_2_ planes are simply separated by Nd layers. In this work, the supercells of size 3 × 3 relative to the primitive P4/mmm cell were employed to explore the effect of Sr ions. Four different models were built with the increasing Sr substitution for Nd ions. 

First-principle density functional theory [54,55] calculations were carried out using the plane-wave pseudopotential code Vienna Ab-initio Simulation Package (VASP) [56]. Blöchl’s projector augmented wave (PAW) method [57,58] and the generalized gradient approximation of Perdew, Burke, and Ernzerhof (GGA-PBE) [59] were employed. Within the PAW, the Nd (5*s*^2^6*s*^2^5*p*^6^5*d*^1^), Sr (4*s*^2^5*s*^2^4*p*^6^), Ni (3*d*^9^4*s*^1^), and O (2*s*^2^2*p*^4^) electrons were treated as valence electrons. Fully relaxed configurations namely, both lattice parameters and atomic positions were optimized using the Quasi-Newton optimization scheme. Architectures were relaxed until the Feynman-Hellman force on each atom was less than 0.01 eV Å^−1^. A fairly robust mixture of the blocked Davidson and RMM-DIIS iteration schemes was used for the electronic minimization algorithm during the geometry optimization [60]. Moreover, the screened exchange hybrid density functional based on the Heyd−Scuseria−Ernzerhof (HSE06) method was adopted to get the exact magnetic and electronic properties [61]. Meanwhile, the iterative matrix diagonalization algorithm which updates all orbitals simultaneously was employed for the static calculations [62,63]. Convergence threshold for the self-consistent field (SCF) total energy was 10^−5^ eV. For both the geometry relaxation and the static calculations, a Monkhorst-Pack grid of 1 × 1 × 4 was used to sample the first Brillouin zone of K space. The kinetic energy cutoff for the plane-wave basis set was 630 eV. Both of them had been carefully checked. Spin polarized calculations with full relaxation of spins were performed. It should be mentioned that non-magnetic calculations as well as improper initial magnetic moments for spin-polarized calculation hardly converge.

## 4. Conclusions

To understand the influence of doping Sr atoms, magnetism and electronic structures of four infinite-layer Nd_9-*n*_Sr*_n_*Ni_9_O_18_ (*n* = 0–2) unit cells were studied with the HSE06 method. Geometries and formation energies as well as the spin densities, *site*- and *lm*-projected DOS, spin-polarized band structures, and Bader charges were calculated.

It was found that the *c* axis of the unit cells enlarged with the increasing Sr atoms, while the *a* and *b* axis stayed almost invariable. That is, doping of the Sr atoms influenced primarily the layer-space of the complexes. Stabilities of the complexes decrease with the increasing Sr atoms. The alignment of the Sr atoms also influenced the stabilities. The two Sr atoms are more prone to aligning along the diagonal line instead of the parallel line of the lattice. However, the parallel one has gotten a smaller magnetic moment than the diagonal one.

Magnetic moments of all the considered models arranged themselves in the two-dimensional NiO_2_ planes and mainly localized on the Ni atoms. The total magnetic moment of the Nd_9_Ni_9_O_18_ shrinks apparently with the Sr substitution for Nd atoms. According to the spin density distributions, the magnetic disordering of the Ni atoms is responsible for the magnetism decrease. While, from a perspective of spin-polarized band structures, it showed that the symmetry of the spin-up and spin-down energy bands around the Fermi levels also influence the total magnetic moments. 

Considering the DOS and energy bands comprehensively, band couple primarily act as the hybridization among the $\{\mathrm{Ni},\mathrm{Nd}\}\text{-}(d_{z^{2}})$, $\mathrm{Ni}\;(d_{x^{2}{-y}^{2}})$, and $\mathrm{Ni}(d_{xz},d_{yz})$ orbitals near the Fermi levels. $\mathrm{Ni}\;(d_{x^{2}{-y}^{2}})$ is the main orbital intersecting the Fermi level. Energy order is generally arranged as follows: $\{\mathrm{Ni},\mathrm{Nd}\}\text{-}(d_{z^{2}})>\mathrm{Ni}(d_{x^{2}-y^{2}})>\mathrm{Ni}(d_{xz},d_{yz})$. Meanwhile, it showed that electrons of Sr atoms tend to locate locally and hybridize weakly with the O atoms. They primarily help to build the infinite-layer structures, and influence the electronic structure near the Fermi level indirectly. It results in the uplift of $\mathrm{Ni}\;(d_{x^{2}{-y}^{2}})$ and $O\;\left(p_{x},\;p_{y}\right)$ orbitals around the Fermi levels. Bader charge analysis indicated that the average charge of the Ni atom is approximately +1. Both the Ni and O atoms lose electrons with the increasing substitution of Sr atoms. This study is useful for understanding the mechanism of high-temperature superconductivity. The progress in comprehending the mechanism of superconductivity may surge with the aim of conceiving potential room-temperature superconductors.

## Figures and Tables

**Table 1:** Average Bader charges for the atoms in Nd_9-*n*_Sr*_n_*Ni_9_O_18_ (*n* = 0–2) models. The two values in the parentheses represent the minimums and maximums.

	Nd	Sr	Ni	O
Nd_9_Ni_9_O_18_	8.93 (8.93, 8.93)		9.29 (9.29, 9.29)	7.39 (7.39, 7.39)
Nd_8_SrNi_9_O_18_	8.89 (8.89, 8.90)	8.40 (8.40, 8.40)	9.26 (9.26, 9.29)	7.39 (7.37, 7.40)
Nd_7_Sr_2_Ni_9_O_18_-Par	9.01 (8.83, 9.14)	8.43 (8.43, 8.43)	9.18 (9.13, 9.20)	7.36 (7.32, 7.39)
Nd_7_Sr_2_Ni_9_O_18_-Dia	9.07 (8.87, 9.35)	8.55 (8.55, 8.55)	9.16 (9.08, 9.23)	7.33 (7.31, 7.35)
Isolated atom	11	10	10	6

## Data Availability

Not applicable.
